# Regulatory roles of miRNAs associated with the aging pathway in tree vegetative phase changes

**DOI:** 10.48130/FR-2023-0009

**Published:** 2023-04-18

**Authors:** Ying Li, Tingting Chen, Wasif Ullah Khan, Xinmin An

**Affiliations:** State Key Laboratory of Tree Genetics and Breeding, National Engineering Research Center of Tree Breeding and Ecological Restoration, Beijing Advanced Innovation Center for Tree Breeding by Molecular Design, College of Biological Sciences and Technology, Beijing Forestry University, Beijing 100083, China

**Keywords:** Flowering regulation, Forest tree, miR156, miR172

## Abstract

The transition from the vegetative juvenile phase to the adult phase is a crucial event in the life cycle of flowering plants, with flowering being the most important milestone. While the regulatory pathways of flowering have been well established in model plants such as *Arabidopsis* and a few crops, the flowering regulation pathways in perennial forest trees remain poorly understood. This paper summarizes the regulation of flowering time by miR156 and miR172, which are the main members of the aging pathway, and also presents new information on the role of miR159 and miR169. These two microRNAs interact with miR156 and miR172 to jointly regulate flowering time in forest trees. Overall, this review sheds light on the complex regulatory mechanisms underlying flowering time in forest trees and provides insights into potential targets for manipulating the flowering time of these economically and ecologically important species.

## Introduction

**Figure 1:**
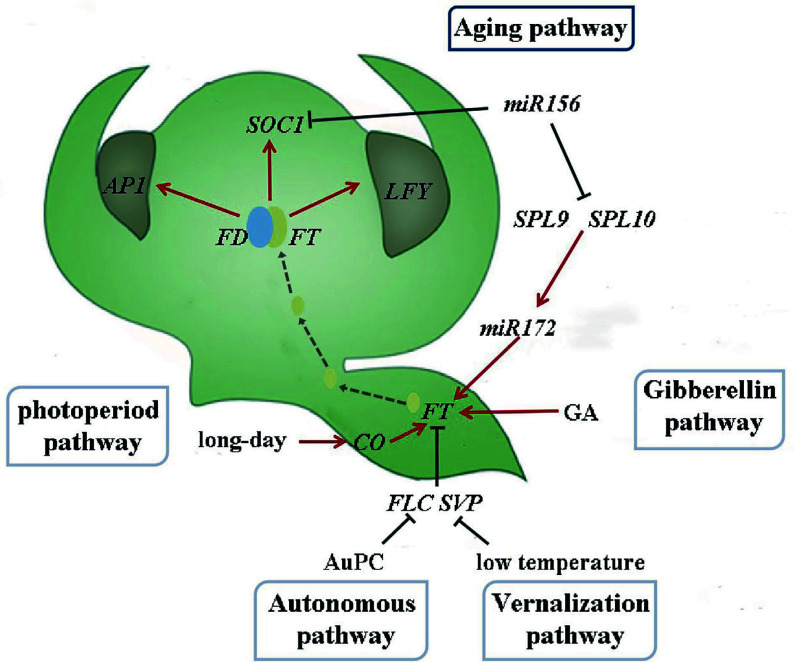
Flowering induction pathway in *Arabidopsis thaliana* (drawing from reference^[12]^).

Plants undergo various developmental transitions throughout their life cycle^[1]^, with the transition from the juvenile to adult phase being particularly significant for flowering plants, where the process of flowering serves as a crucial indicator^[2]^. Flower development is vital for plant growth and reproduction. The regulatory mechanism of plant flowering is intricate and involves a complex network. The photoperiod, vernalization, autonomous, gibberellin, and aging pathways are the five established pathways for flowering induction in model plants such as *Arabidopsis thaliana* (Fig. 1)^[3−7]^. Each pathway regulates gene expression through distinct molecular mechanisms that can interact with one another, culminating in a vast and intricate regulatory network.

Plants undergo a vegetative growth phase until they attain a specific age before they can flower. The aging pathway provides an internal developmental signal that prevents premature flowering and promotes flowering during the adult stage without the need for external factors^[8]^. This pathway interacts with the photoperiodic, gibberellin, and vernalization pathways^[9−11]^, ultimately regulating plant flowering *via* the flowering integrator.

MicroRNAs (miRNAs) are short, non-coding endogenous RNA molecules that are approximately 18−22 nucleotides in length and play a significant role in plant development and gene regulation^[13,14]^. Through translational repression or transcriptional cleavage of target genes, miRNAs can repress the expression of target genes, thereby regulating various processes of plant growth and development^[15,16]^. In recent years, the aging pathway has garnered increasing attention for its role in regulating plant flowering, and miRNAs have been found to control the aging pathway-mediated vegetative phase change^[17]^. miR156 and miR172 are the central regulatory hub of the aging pathway. They were the first molecular markers discovered in *Arabidopsis* that respond to aging pathway regulation^[18]^ and have been systematically studied in species such as *Zea mays* and *Chrysanthemum morifolium*, providing an important approach to explore and reveal the regulatory mechanisms of plant growth and development.

Currently, the miRNA156 and miRNA172-mediated aging pathway has been extensively studied in model plants, but there is limited research on perennial trees. Perennial trees are characterized by a long juvenile phase and delayed flowering and fruiting, which significantly impacts their reproductive efficiency and limits the breeding process. Therefore, it is necessary to investigate the age-regulated pathways in trees. This review primarily focuses on the role of miRNA156 and miRNA172 in vegetative phase transition in trees and discusses the involvement of other miRNAs outside the aging pathway. This review also covers the flowering-related miRNAs and their target genes, as well as the mode of action between miRNAs.

## Molecular mechanism of miR156 and miR172 in aging pathway

### Molecular mechanism of miRNA156

miR156 is a highly conserved and widely studied member of the plant miRNA family. It was first discovered in *Arabidopsis*^[19]^ and is involved in regulating plant phenotypic changes and flowering through its interaction with the *SPL* family^[19,20]^. The 3' UTR of *SPL* family members contains a functional miRNA response element (MRE), which can be targeted by miR156 to promote degradation or repression of translation of target genes^[21]^.

Expression of miR156 is high during the seedling stage and gradually decreases as the plant develops^[22]^. In *Arabidopsis*, the miR156 family consists of eight members, with miR156a and miR156c playing a dominant role in vegetative phase change^[23]^. The importance of miR156 in flowering is demonstrated by the delayed flowering phenotype resulting from miR156 overexpression^[24]^. Loss of function of miR156 in STTM (short tandem target mimic) lines in *Arabidopsis* leads to an early flowering phenotype, with upregulation of miR172, *SPL*, and some flowering activators in the STTM-miR156 lines^[25]^.

A total of 17 *SPL* genes were identified in *Arabidopsis,* 11 of which are direct downstream target genes of miR156^[21]^. Currently, the miR156-*SPL* module was found to affect the flowering process in plants mainly through the regulation of downstream related flowering genes. There are two main pathways through which miR156 regulates flowering time in *Arabidopsis*: first, in SAM (shoot apical meristem), the target genes of miR156, *SPL3* and *SPL9*, induce flowering by activating floral organ characteristic genes such as *SOC1* and *AP1*. The other pathway is in leaves, where *SPL9* activates the expression of miR172 genes and further suppresses the expression of flowering repressors such as *AP2* to accelerate flowering^[12]^.

### Molecular mechanism of miR172

miR172, a downstream target of miR156, plays a crucial role in the transition to the adult phase in plants^[26]^. This miRNA is highly conserved in ferns, gymnosperms, and angiosperms^[27]^. Multiple studies have shown that miR172 regulates flowering time, floral organ development, and plays a crucial role in vegetative phase change in several species^[28]^. In *Arabidopsis*, the precursor of miR172 is encoded by miR172a-miR172e. Overexpression of miR172 in *Arabidopsis* has been found to promote the transition from the juvenile to adult phase^[29−31]^.

The expression of plant miR172 increases during growth and development, while the expression of its target genes decreases, indicating that miR172 primarily functions by repressing translation of its targets^[32]^. Its targets include *AP2/ERF* transcription factors^[33]^, with six target genes identified in *Arabidopsis*: *AP2*, *SNZ*, *SMZ*, *TOE1*, *TOE2*, and *TOE3*^[34]^. Through interaction with the conserved structural domains of these target genes, miR172 promotes flower development by suppressing their accumulation^[35]^.

**Figure 2:**
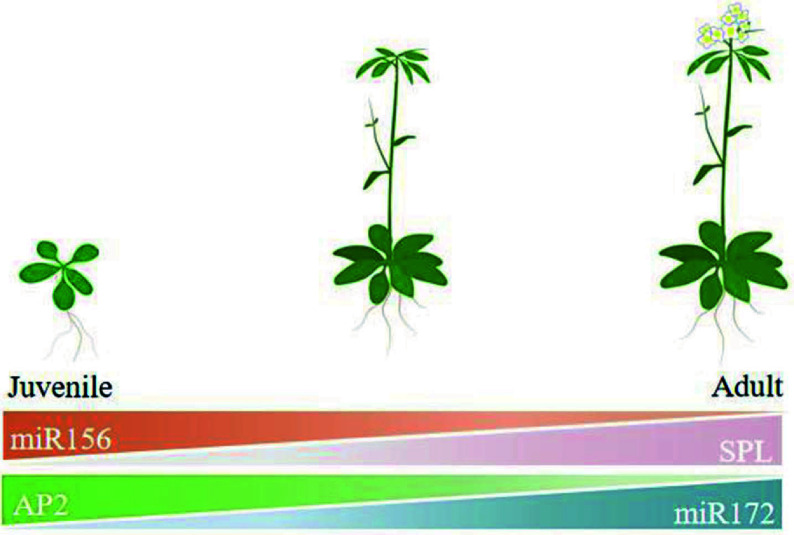
Vegetative phase change mediated by miR156-miR172 in *Arabidopsis thaliana* (drawn from reference^[12]^).

In model plants such as *Arabidopsis*, the miR156-*SPL*-miR172-*AP2* pathway is well understood (Fig. 2). The miR156 gene family indirectly regulates miR172 expression by suppressing *SPL* gene expression, which in turn reduces the transcription of downstream genes like *AP*2 and *TOE*. This regulatory pathway plays a crucial role in all plant development processes^[36]^.

## The roles of miR156 and miR172 in flowering regulation in trees

### The role of miR156 and its target gene *SPL* in trees

Plants exhibit distinct morphological features during various developmental stages. For instance, *Arabidopsis* shows differences in leaf morphology between juvenile and adult stages, with adult leaves having serrations and abaxial trichomes^[37]^. In contrast, perennial woody plants with longer developmental cycles display subtle differences between their juvenile and adult stages. In such plants, the vegetative phase change has been studied by examining phenotypic characteristics such as leaf morphology, stem morphology, and branching. Leaf morphology was first investigated as early as 1990, revealing that plants displayed varying phenotypic features and sizes, photosynthetic efficiency, or epidermal characteristics at different developmental stages^[38]^. Recent studies have also examined the vegetative phase change in poplar^[39]^ and apple^[40]^ species, and investigated the developmental status of trees at the stem^[41]^ and branches^[42]^.

In some heteroblastic species, including *Eucalyptus globulus*, *Acacia confusa*, *Quercus acutissima*, and *Acacia colei*, the transition from juvenile to adult phases is marked by distinct morphological changes. For instance, *Acacia* trees shift from pinnate compound leaves to produce simple undivided leaves known as phyllodes. The expression patterns of miR156 in juvenile and adult leaves of these species were analyzed separately, and it was found that miR156 expression decreased with age, consistent with the pattern observed in *Arabidopsis*. Additionally, miR156 expression levels correlated with changes in leaf morphology and internode distance, indicating that miR156 is associated with the vegetative phase change. Overexpression of miR156 in *Populus canadensis* suppressed the expression of *SPL* and miR172 in transgenic lines and significantly prolonged the juvenile period, further supporting the role of miR156 in phase change^[43]^. Similarly, in *Populus tremula × P. alba*, overexpression of *Cg1*, a maize endogenous gene encoding miR156, resulted in significant branching and reduced lignin content in transgenic lines. Furthermore, a positive correlation was observed between the severity of the phenotypic changes and *Cg1* expression levels, underscoring the significant effects of miR156 on poplar growth and development^[44]^. A study investigating miR156-*SPL* expression regulation patterns in *Populus tremula* *×* *P. alba* found that lower miR156 expression levels in leaves of different node positions were associated with smaller leaf length-width ratios, while transgenic lines with high miR156 expression levels had smaller leaf areas and shorter petiole lengths, further confirming the role of miR156 in the vegetative phase change of poplar^[45]^.

Research on various plant species including *Morus alba*, *Jatropha curcas*, *Citrus* *reticulata*, *Castanea mollissima, Punica granatum,* and *Cunninghamia lanceolata* has confirmed the existence of the miR156-*SPL* regulatory network which plays an important role in plant vegetative phase change. In mulberry, miR156 expression gradually decreases with age while *SPL* and miR172 expression show the opposite trend. Studies have shown that the miR156-*SPL*-miR172 regulatory network is present in mulberry and can activate the expression of miR172a by recognizing the GTAC *cis* element in the promoter region^[46]^. In *Jatropha curcas*, *JcSPL3* plays an important role in regulating age development, with its expression level increasing with plant age. Overexpression of *JcSPL3* in *Arabidopsis* results in earlier flowering, validating its function^[47]^. Similarly, in *Citrus reticulata*, *CiSPL5* plays a major role in promoting flowering and accelerating the juvenile process, regulated by miR156^[48]^. Genome-wide identification of the *SPL* gene family in *Castanea mollissima*^[49]^, *Punica granatum*^[50]^, and *Cunninghamia lanceolata*^[51]^ further supports the importance of the miR156-*SPL* regulatory model in plant vegetative phase change.

As *Populus euphratica* leaves matured, they transitioned from a lanceolate to a broad-ovate shape, and this process was associated with a decrease in miR156 expression and an increase in the expression of 12 *SPLs*, suggesting that the miR156-*SPL* regulatory pathway plays an important role in the plant's vegetative phase change^[52]^. Similarly, in *Eucalyptus globulus*, differences in miR156 expression were found to underlie the timing of the plant's vegetative phase change, as indicated by QTL analysis of an outcross F2 family^[53]^. In studies of *Persea americana*, *Mangifera indica*, and *Macadamia integrifolia*, miR156 expression gradually decreased with tree age, and the miR156-*SPL3/4/5* pattern was found to be conserved in all three species, consistent with previous studies^[54]^.

The aging pathway has been investigated in gymnosperms. In *Ginkgo biloba,* miRNA expression changes at different ages were analyzed, revealing that miR156 was highly expressed in older individuals, and its target genes did not vary with age, unlike the miR156 expression pattern observed in angiosperms^[55]^. In *Pinus tabuliformis*, 26 miRNA families and 74 targets were identified through small RNA sequencing and parallel analysis of RNA ends (PARE). Among these miRNAs, miR156-*SPL*, which is involved in angiosperm reproductive development, was also found to be present in *P. tabuliformis*^[56]^. *Picea abies* genome analysis identified 11 *SPL* genes, *PaSPL1-11*, among which *PaSPL1, PaSPL2, PaSPL10, and PaSPL11* have miR156 binding sites. The evolutionary relationships suggested that *PaSPL1, PaSPL10,* and *PaSPL11* play a crucial role in reproductive stage change, but miR156-*SPL* expression patterns vary in different tissues. Additionally, mutations in the miR156 binding site of the *PaSPL1* gene were found to cause early flowering of the *acrocona* mutant, demonstrating the importance of the aging pathway in regulating reproductive phase change in *P. abies*^[57]^. These studies show that miR156 and its target gene *SPLs* are conserved in gymnosperms, but no age-related expression pattern of miR156-*SPL* was observed in gymnosperms, suggesting that there is no trend of decreasing or increasing expression levels of miR156 or *SPL* with increasing plant age in gymnosperms.

**Figure 3:**
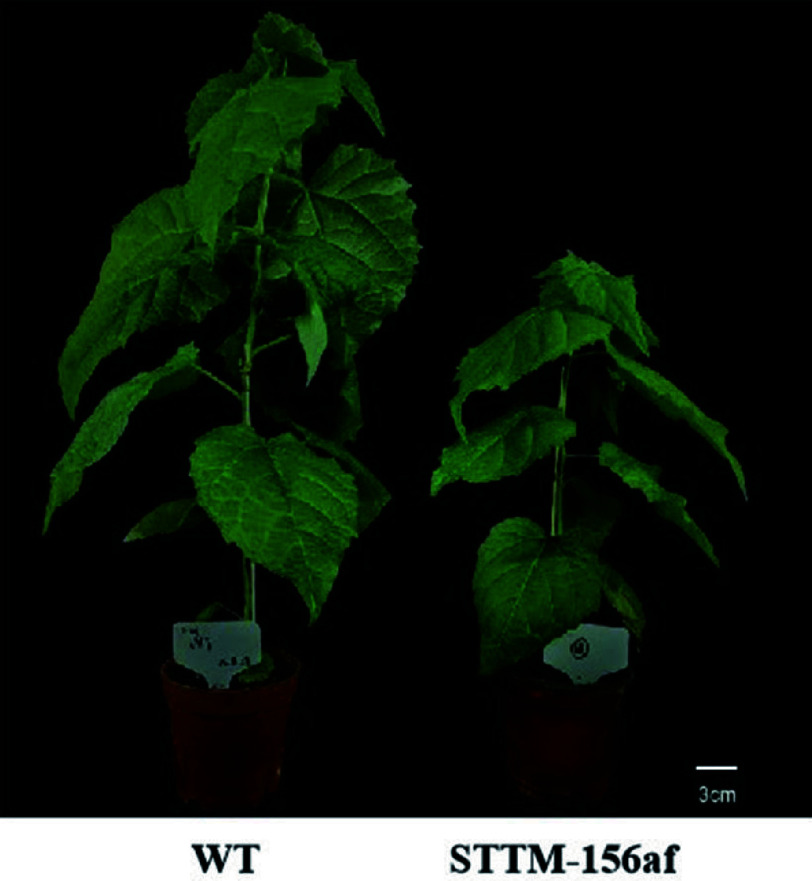
STTM-miR156af interference expression transgenic plants.

Our team studied the precursor and mature sequences of the miR156 gene family using the *Populus tomentosa* genome data, and we were able to clone 11 members of the family, which we designated pto-miR156a/b/c/d/e/f/g/h/i/j/k based on comparison results. After that, we analyzed the family members' sequences, predicted secondary structure and target genes, and examined their expression patterns. We created an expression interference vector for STTM-miR156af based on the mature sequence's conserved domain. We identified DNA and RNA levels in transgenic lines produced by genetic transformation mediated by Agrobacterium. Our study revealed that the transgenic lines exhibited a significant reduction in the transcription of miR156a/b/c/d/e/f when compared to the wild type. Additionally, we found that the plant height of the transgenic lines was 35.44% shorter than that of the wild type (Fig. 3), indicating that miR156 plays a crucial role in plant height regulation. These findings are in line with previous studies that utilized STTM technology to repress miR156a in poplar^[45]^ and observed a decrease in plant height in the transgenic line relative to the wild type.

To gain further insights into the regulatory mechanism of miR156 in the aging pathway of poplar, we plan to conduct statistical analysis of related phenotypes in leaves and other aspects. Furthermore, we intend to integrate sequencing data to explore the complex interplay between miR156 and aging-related genes in poplar. Our study will provide a comprehensive understanding of the role of miR156 in regulating plant growth and aging and offer potential targets for tree improvement.

### The role of miR172 and its target gene AP2-like in trees

Changes in miR172 were observed in the leaves of various plants such as *E. globulus*, *A. confusa*, *Q. acutissima*, and *A. colei*^[43]^. Unlike miR156, miR172's expression increased with age and was linked to leaf morphology and internode distance, indicating its role in regulating the vegetative phase change. Overexpression of miR156 in *P. canadensis* suppressed the expression of miR172, prolonging its juvenile phase. Conversely, miR172's expression gradually increased with age in poplar, indicating its role in regulating the vegetative phase change^[43]^. In mulberry, miR172's expression was found to be proportional to the tree's age, and six miR156 target genes activated miR172 expression^[46]^. The study of miR172 in *J. curcas* revealed that its expression increased with tree age, and its overexpression caused early flower and leaf morphological changes, while suppressing the expression of miR172 target genes^[58]^. In *Magnolia* *×* *soulangeana* ‘*Changchun*’, miR172 expression levels were significantly upregulated during the transition from vegetative to reproductive growth, and its target genes *MsAP2* and *MsTOE3* were downregulated, indicating its significant role in the vegetative phase change of magnolia through molecular regulation^[59]^.

In more mature tissues of avocado (*Butyrospermum parkii*), miR156 was found to be more abundant, while miR172 was less abundant compared to young tissues. The expression of their target genes, *PaSPL4* and *PARA2.7B*, respectively, was found to be negatively correlated with the abundance of miR156 and miR172. These findings suggest the conservation of the miR156-*SPL*-miR172 pathway in avocado^[60]^. However, a different study showed that although miR172 abundance significantly increased during the transition from juvenile to adult avocado, the expression of *AP2*-like genes did not decrease with increasing miR172 abundance. As this study was based on RNA transcript abundance, it cannot conclusively rule out the possibility of a translational repressive effect of miR172^[54]^.

The expression of miR172 and its target gene *AP2* was investigated in different age groups of *Ginkgo biloba*, a gymnosperm. The study showed that unlike in angiosperms, miR172 and its target genes did not exhibit an age-related differential expression pattern^[55]^. In *Pinus tabuliformis*, miR172-*AP2* was also identified through small RNA sequencing and parallel analysis of RNA ends (PARE) in both female and male cones^[56]^. Homologs of *AP2* in *Arabidopsis*, *PaAP2L1, PaAP2L2*, and *PaAP2L3*, were identified in *Picea abies,* and it was confirmed that all three genes contained sequence motifs complementary to miR172. The overexpression of *PaAP2L2* in *Arabidopsis* resulted in delayed development in transgenic lines, supporting the conservation of *AP2* in *Picea abies*^[61]^. In conclusion, miR172-*AP2* is conserved in gymnosperms, but unlike in angiosperms, no clear expression pattern was observed.

**Figure 4:**
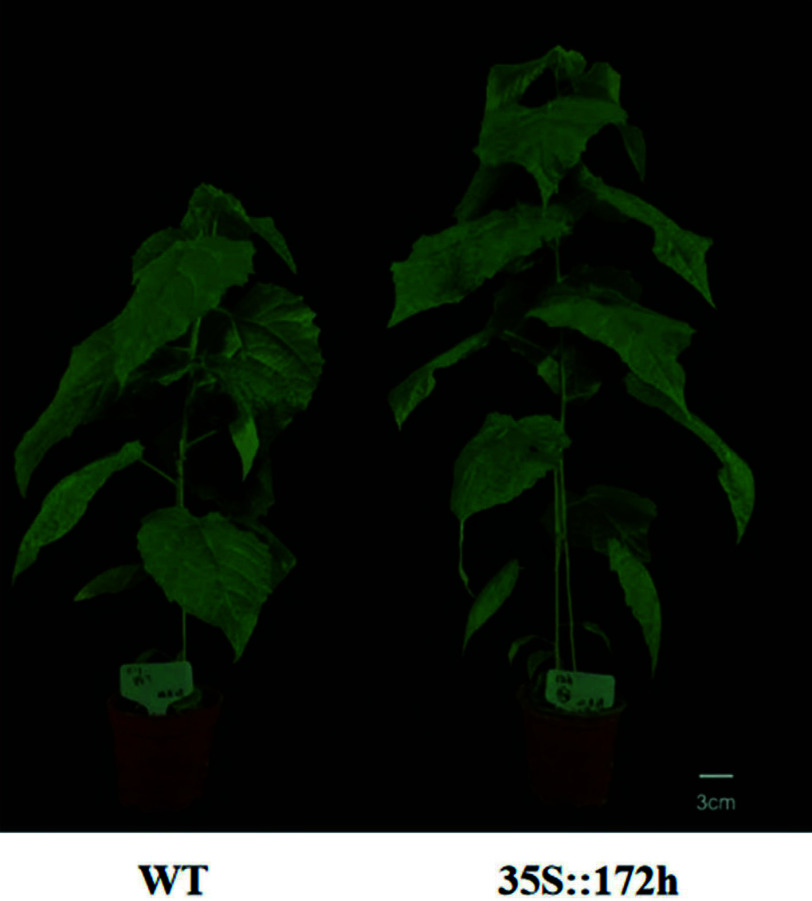
MIR172h overexpression transgenic plants.

Using genome data from *P. tomentosa*, our group analyzed the precursor and mature sequences of the miR172 gene family. We identified seven members of the miR172 family, which we named Pto-miR172a/b/c/e/g/h/i based on comparative analysis. We conducted sequence and expression pattern analyses of these seven members and selected key members to construct a C6-MIR172h overexpression vector. We then carried out genetic transformation in poplar and observed a 26.82% increase in height growth in the transgenic line compared to the wild type (Fig. 4). These results are consistent with previous findings on the role of miR172 in regulating plant height in cotton^[62]^ and rice^[63]^. We will further analyze the phenotypic data of the transgenic lines and explore the mechanisms of miR172 in combination with transcriptomic data. This will provide a basis for elucidating the aging pathway of poplar flowering regulation.

## Other miRNA-mediated regulatory pathways

In addition to miR156 and miR172, other miRNAs involved in regulating flowering have been reported in recent years, including miR159 and miR169. While these miRNAs are not part of the aging pathway, they interact with miR156/172 to jointly regulate plant flowering.

### The miR159-Regulated Pathway

miR159 is a highly conserved microRNA family that is commonly found in plants^[64]^. Studies have shown that miR159 targets a class of *GAMYB* genes that encode *R2R3-MYB* transcription factors. These *GAMYB* genes activate downstream genes related to GA signaling and are also referred to as '*GAMYB'* genes^[65]^. In *Arabidopsis thaliana*, miR159 regulates the timing of the vegetative phase change by suppressing the expression of *MYB33*, preventing it from hyperactivating miR156 expression during the seedling stage^[66]^.

The mechanism of miR159 action has also been explored in trees in recent years. The expression of miR159a increased significantly during the dormancy transition in *Malus pumila*, whereas the expression of its putative target genes *MdMYB33* and *MdMYB65* decreased substantially during this period, which also suggests that miR159-*MYB* plays an important role during dormancy in *M. pumila*, regulating *M. pumila* break dormancy to further promote floral bud differentiation^[67]^. Although *PtrMYB012* contains a conserved miR159 target sequence, it is not a target of *Arabidopsis* miR159. Therefore, its transcript is not degraded and overexpression of *PtrMYB012* in *Arabidopsis* leads to phenotypes such as upwardly curled rosette leaves and plant dwarfism. In contrast, *PtrMYB012* was affected by poplar miR159, resulting in degradation of transcripts, and ultimately no significant phenotypes occurred in transgenic poplar, suggesting that miR159-*MYB* in poplar regulates plant development during the vegetative phase^[68]^. miR159 is one of the most abundantly expressed miRNAs during different developmental periods in *Taxus chinensis*, and studies have demonstrated that it effectively represses *GAMYB* and thus regulates the growth and development of *T. chinensis*^[69]^.

### The miR169-regulated pathway

Currently, miR169, which is the largest miRNA gene family in plants, has been extensively studied in relation to plant growth and development, resistance, and regulation of flowering^[70−72]^. The *NFYA* family is the most important target of miR169. The miR169-*NFYA* module also plays a crucial role in the development of floral organs in plants. In *Arabidopsis*, *AtNFYA2* binds to and activates the expression of the AACCT component of the flowering repressor *FLC* (flowering locus C) promoter, thus delaying the flowering time of plants^[71]^. Similarly, the binding of *Arabidopsis* *NFYA8* to the miR156 promoter prevents the transition of plant seedlings to adults and delays the flowering time^[73]^.

The role of miR169 in regulating flowering has been investigated in *Populus tremuloides*. The study found a negative correlation between the expression dynamics of ptr-miR169a and its target gene *PtrHAP2-5*, particularly at the beginning of dormancy when ptr-miR169a expression increased and *PtrHAP2-5* transcript levels decreased. The study also verified that the transcriptional decline of *PtrHAP2-5* was caused by cleavage at the predicted miR169a site. It was suggested that *HAP2* has an opposite effect on *FT* transcriptional activity. Moreover, miR169 was found to alleviate the repression of the *PtrFT* gene during dormancy by down-regulating the abundance of *PtrHAP2* transcripts, thus promoting plant flowering^[74]^.

## Conclusions and future prospects

This review critically examines recent research on miRNAs and their roles in regulating the flowering of trees, with a particular focus on miR156 and miR172 in the aging pathway, along with miR159 and miR169, which interact with the aging pathway to promote flowering (as outlined in Table 1). Based on the analysis of the findings, it was observed that miR156 and miR172 are commonly present in angiosperms, and their target genes exhibit either an increase or decrease in expression as the plant ages. However, there is no evidence to support age-related expression patterns for these miRNAs in angiosperms.

**Table 1:** miRNAs that regulate flowering time in trees and their target genes.

miRNA	Target genes	Species	References
miR156	*SPL3*,*4*,*5*,*9*,*10*	*Arabidopsis thaliana*	[12, 17, 21, 22]
	*EglSPL3*,*9*	*Eucalyptus globulus*	[43]
	*PcSPL3*,*9*	*Populus canadensis*	
	*PtSPL23*,*24*	*Populus tremula × alba*	[45]
	*MnSPL2*,*8*,*10A*,*10B*, *15*,*16A*	*Morus alba*	[46]
	*JcSPL2*,*3*,*4*,*5*,*6*,*9*,*10*, *11*,*13*,*16*	*Jatropha curcas*	[47]
	*CiSPL5*	*Citrus reticulata*	[48]
	*CmSPL2*,*4*,*5*,*6*,*9*,*10*,*11*,*13*,*16*,*17*	*Castanea mollissima*	[49]
	*PgSPL1*,*2*,*3*,*6*,*7*,*11*,*12*, *13*,*14*,*15*	*Punica granatum*	[50]
	*Unigene2030*,*2872*	*Cunninghamia lanceolata*	[51]
	*PeuSPL4*,*9*	*Populus euphratica*	[52]
	*EglSPL3*	*Eucalyptus globulus*	[53]
	*PaSPL4*,*9a*,*9b*	*Persea americana*	[54]
	*MiSPL3*,*4*,*5*	*Mangifera indica*	
	*MciSPL4*	*Macadamia integrifolia*	
			
	*PtSPL1*,*3*	*Pinus tabuliformis*	[56]
	*PaSPL1*,*2*,*10*,*11*	*Picea abies*	[57]
miR172	*AP2*;*SNZ*;*SMZ*;*TOE1*,*2*,*3*	*Arabidopsis thaliana*	[33, 35]
	*PaAP2*,*2.7a*,*2.7b*	*Butyrospermum parkii*	[54]
	*MiAP2*,*2.7a*,*2.7b*	*Mangifera indica*	
	*MciAP2*	*Macadamia integrifolia*	
	*JcAP2*;*JcTOE1*,*2*,*3*	*Jatropha curcas*	[58]
	*MsAP2*; *MsTOE3*	*Magnolia × soulangeana ‘Changchun’*	[59]
	*PtAP2L2*,*3*	*Pinus tabuliformis*	[56]
	*PaAP2L1*,*3*	*Picea abies*	[61]
miR159	*GAMYB*	*Arabidopsis thaliana*	[65]
	*MdMYB33*,*65*	*Malus pumila*	[67]
	*PtrMYB012*	*Populus alba × P. tremula*	[68]
	*GAMYB*	*Taxus chinensis*	[69]
miR169	*NFYA*	*Arabidopsis thaliana*	[71]
	*PtrHAP2–5*	*Populus tremuloides*	[74]

Currently, our understanding of the regulatory mechanisms of flowering in plants has largely been acquired from annual model plants. However, the flowering process in perennial trees is more complex due to seasonality and annual cycles, which may involve additional and different regulatory mechanisms. Unfortunately, research on elucidating flowering mechanisms in perennial plants has progressed slowly due to complications arising from their perennial growth and the recurring nature of the flowering process. Although studies on flowering mechanisms have been carried out in some perennial horticultural plants or crops, these studies have provided genetic resources and cases for flowering studies in perennials, deepening our understanding of flowering mechanisms. Despite this progress, relatively few studies have focused on the molecular mechanisms of flowering regulation in tall tree species, and age-regulated pathways have barely been reported. miRNAs are known to play an important role in regulating flowering as well as overall growth and development in plants. Therefore, understanding the molecular mechanisms of miRNA action in perennial trees holds great research and practical value. It is necessary to deepen our research on the mechanisms of miRNA action in trees to advance the breeding process.
